# Cross-protective immunity from inactivated whole cell *Rickettsia rickettsii* antigen vaccine prevents fatal infection across heterologous strains

**DOI:** 10.21203/rs.3.rs-10505079/v1

**Published:** 2026-08-10

**Authors:** Perle Latré de Laté, Ian M. Stoll, Jonathan Ferm, F. Liliana Crosby, Dominica Ferm, Deepika Chauhan, Huitao Liu, Suhasini Ganta, Dae Y. Kim, Jodi L. McGill, Roman R. Ganta

**Affiliations:** 1 Department of Pathobiology and Integrative Biomedical Sciences, College of Veterinary Medicine, University of Missouri, Columbia, MO; 2 Veterinary Diagnostic Laboratory division, Department of Pathobiology and Integrative Biomedical Sciences, College of Veterinary Medicine, University of Missouri, Columbia, MO; 3 Department of Veterinary Microbiology and Preventive Medicine, Iowa State University, Ames, IA

## Abstract

Tick-borne *Rickettsia rickettsii* causes fatal Rocky Mountain spotted fever (RMSF) in humans and dogs. Canine vaccination represents a strategic approach to improve companion animal health while simultaneously reducing the broader public health burden of RMSF. In this study, we evaluated the pathogenicity of three distinct *Rickettsia rickettsii* strains; Iowa, Sheila Smith, and Morgan, in dogs and assessed the protective efficacy of whole-cell antigen-based vaccines (WCAVs) derived from these strains. While the Iowa strain caused only mild clinical signs, the Sheila Smith and Morgan strains induced severe clinical disease. Formulated WCAVs demonstrated robust protective efficacy, entirely eliminating mortality and severe clinical illness across all challenged groups. Vaccinated animals exhibited significantly reduced bacteremia, minimized tissue damage, and stabilized systemic biochemical markers. This robust protection correlated with strong humoral responses and antigen-specific CD4^+^ and CD8^+^ T-cell proliferation. These findings establish a foundational framework for developing safe, effective vaccine against fatal RMSF.

## INTRODUCTION:

Rocky Mountain spotted fever (RMSF) is a potentially fatal tick-borne disease affecting humans and dogs. RMSF is the result of infection with the obligate intracellular bacterium *Rickettsia rickettsii* and is frequently reported across various parts of North, Central, and South America^1–8^. The high incidence of the disease with high mortality rates are reported from parts of Mexico and in certain Indian reservations of the USA in recent years^2,3,9,10^. The bacterium is transmitted by several species of hard ticks, including *Dermacentor* species (*D. variabilis* and *D. andersoni*), *Rhipicephalus sanguineus* sensu lato and *Amblyomma* species, such as *A. americanum, A. aureolatum* and *A. cajennense* complex with reported regional variations in vector predominance and the disease severity^11–15^.

Early clinical manifestations of RMSF often include elevated body temperature, headache, lethargy, nausea/vomiting, Myalgia, stomach pain and abdominal palpations, lack of appetite and petechial rashes initially appearing on the wrists and ankles which gradually spread to other parts of the body^16–18^. These clinical signs can overlap with other tick-borne and viral illnesses leading to complications of accurate and timely diagnosis and treatment of the disease^4,5,19,20^. Following tick transmission, *R. rickettsii* invades vascular endothelial cells, disseminates cell-to-cell by exploiting the host actin by polymerizing it to form comet-like tails which help its rapid movement through cytoplasm and into membrane protrusions. This results in systemic vasculitis through endothelial cell damage and increased vascular permeability; vasculitis affects major organs such as brain, lungs, liver, testes in males, and kidneys^21–24^.

Genomic and phenotypic diversity among *R. rickettsii* strains correlates with differences in virulence across animal models and the level of disease severity in humans^25–29^. Sheila Smith strain, belonging to the virulent western lineage and Morgan strain representing a distinct lineage, both of which differ markedly from the less virulent Iowa strain in terms of pathogenicity and molecular features, including outer membrane protein processing^28,29^. Strain-specific differences have been associated with variable clinical outcomes as well as immune response profiles^28^ suggesting that vaccine efficacy may be strain dependent.

Currently, the recommended antibiotic treatment for *R. rickettsii* infections is doxycycline and it is effective only when administered during the first few days following infection^30–32^. If untreated or treatment is delayed, RMSF can quickly progress to life threatening illness resulting in high mortality, particularly in young children and dogs^1–3,6^. In recent years, numerous human RMSF infections with fatality rates ranging from 30–80% have been reported in Mexico, Brazil and parts of the USA^6–8,33^. Despite the discovery of RMSF over 120 years ago, there is currently no vaccine to prevent it. Accurate diagnosis remains a complex challenge due to the broad and non-specific clinical manifestations that often overlap with viral and other viral and other bacterial infections^19,20^, thus an efficacious vaccine that abates the progression of RMSF is of high importance to public health. Subunit vaccine candidates targeting surface proteins, such as OmpA, OmpB, and Adr2 have elicited immune responses in small animal models but generally appeared to provide only partial protection^34–39^. We previously reported that a subunit vaccine constituted of two recombinant proteins, OmpB-4 and Adr2, failed to provide immune protection against virulent *R. rickettsii* infection in the physiologically and clinically relevant canine host^40^. Strategies that generate robust and durable protection in natural hosts remain elusive, underscoring the urgent need for an effective vaccine and new therapies.

In our prior published work, we established a physiologically relevant canine infection model showing that a whole-cell antigen vaccine (WCAV) confers complete protection against mortality when challenged with a homologous strain by inducing strong antigen-specific IgG1 and IgG2 synthesis, as well as expanding CD4^+^ and CD8^+^ T cells responses^40,41^. We further reported similar immune protection in a follow-up study where we documented that the WCAV protection is independent of the adjuvant used^40,41^. Although our prior work demonstrated that a WCAV can confer homologous protection in the canine model of RMSF, its ability to protect against heterologous *R. rickettsii* strains is yet to be established. To address this gap of knowledge, in the current study, we evaluated the protective efficacy of three different WCAV formulations derived from either of the two virulent strains, Sheila Smith and Morgan, or using the avirulent Iowa strain. We demonstrate that whole-cell *R. rickettsii* antigens as WCAV from virulent and avirulent strains elicit robust cross-protection against heterologous virulent challenges in the natural canine host. Unvaccinated controls developed severe disease and 60% mortality following Sheila Smith or Morgan strain challenge, whereas Iowa strain exposure induced only mild morbidity. Protection associated with coordinated expansion of humoral and cellular immunity, providing the first definitive evidence of cross-strain RMSF protection and establishing the immunological framework for this vaccine efficacy.

## RESULTS:

### 1. Virulent Sheila Smith and Morgan strains of *R. rickettsii*, but not the avirulent Iowa strain induced severe RMSF.

Five dogs each representing both sexes per group were infected with one of the three *R. rickettsii* strains: Sheila Smith, Morgan, or Iowa. Comprehensive clinical monitoring throughout the infection period (clinical sign scoring data provided in Table S1) allowed comparison of the disease progression and survival across the three infection groups. Symptom scoring data revealed clear differences in the clinical signs developed by dogs following infections with Sheila Smith and Morgan strains with the severity peaking between days 6 and 11 when compared to the Iowa strain infection during the acute phase of infection. Dogs infected with either of the two virulent strains developed rapidly escalating clinical signs beginning from day 2 which continued until day 14 post infection, with sustained moderate to severe disease development throughout the acute phase and having mean scores of 2 (Fig. 1A). In contrast, Iowa strain-infected dogs had only mild, transient symptoms, with mean scores rarely exceeding 1.

#### 1.1. Clinical signs and severe disease were evident only with virulent strain infections of R. rickettsii.

To evaluate cumulative disease burden, we calculated the area under the curve (AUC) of daily clinical severity scores from day 0 to day 14. Both Sheila Smith- and Morgan-infection groups showed significantly higher AUC values compared to the Iowa-infection group, reflecting greater intensity and duration of the disease with these two strain infections (Fig. 1B; *p < 0.05). Whereas Iowa-infection group dogs displayed uniformly low AUC values, confirming the minimal pathogenicity for this strain, as previously documented with the guinea pig infection model^28^. The survival outcomes mirrored those of clinical scores, with dogs infected with virulent strains exhibiting the most severe outcome requiring euthanasia in 60% of the animals within the first two weeks of infection (Fig 1C). In contrast, all five Iowa-infection group dogs exhibited minimal clinical signs until the study endpoint (40 days post infection) (Fig 1.C). Body temperatures for dogs in all three groups remained stable during the pre-challenge period. In contrast, Sheila Smith- and Morgan-infected dogs developed febrile responses by day 2 with peak temperatures reaching about 105°F and 104.3°F, respectively (Fig. 2A). In contrast, the Iowa group remained normothermic except for a transient, mild increase in body temperature observed on day 2 post-infection. Body weights following infection challenges with Sheila Smith or Morgan strains decreased during acute disease phase, whereas Iowa-infected dogs maintained stable body weight over the entire post-infection assessment period, although the data were not statistically significant due to high variations among the groups, except for day 6 post infection (Fig. 2B).

#### 1.2. Hematological disturbances observed primarily in dogs infected with the two virulent strains of R. rickettsii.

Dogs receiving *R. rickettsii* infection exhibited significant hematological changes when infected with the two virulent strains (Fig. 3). Red blood cell (RBC) counts remained within or near reference ranges across all groups of infected dogs, although Sheila Smith–infection group exhibited a modest reduction at day 3 post-infection (Fig. 3A and B). Lymphocyte counts displayed clearer strain-dependent differences with Sheila Smith- and Morgan-infected groups having marked lymphopenia between days 3 and 9 post-infection, while Iowa-infection resulted in dogs maintaining normal ranges (Fig. 3C and D). Lymphocyte numbers progressively recovered for both the virulent-strain infection groups beyond this time. A downward trend in the monocyte values was evident for all three groups by day 3 post infection with significant differences observed for the Sheila Smith- and Iowa-infected groups, while all animals from all groups had normal values beyond this point (Fig. 3E and F). A similar and rapid decline of eosinophils was observed also for all three groups starting from day 3 post infection and remained significantly lower until day 9, which recovered to nearly normal values beyond this day (Fig. 3G and H). Despite the noted decline, the Iowa strain-infected dogs maintained higher eosinophil values across all days post infection assessment compared to those that received Sheila Smith or Morgan strain infections. Thrombocytopenia was observed for both Sheila Smith- and Morgan-infected groups with a significant drop in platelets between days 3–13 post-infection, whereas the drop in platelets in the Iowa-infected group was less pronounced (Fig. 3I and J). Circulating neutrophils in all infected animals and for all time points were mostly stable except for the Morgan group, in which a significant rise was noted for day 13 post infection. This increase was predominantly for the three dogs that developed severe disease and the increase in neutrophils was evident immediately prior to the humane end point (Fig. 3K and L, Fig S1). Following this time point, the surviving animals in this group had similar neutrophil counts as observed for other control group dogs (Fig. 3K). In the Sheila Smith infection control group, the neutrophil counts were similar to the Iowa infected group despite three dogs developing the severe disease.

Biochemical analyses of plasma samples collected during the study period revealed significant variations in some of the blood chemistry profiles assessed (Fig. 4). Blood urea nitrogen (BUN) and creatinine levels declined significantly on day three post infection in both virulent strain infected animals, while remaining unchanged for the Iowa group (Fig. 4A and B). These changes were also evident when BUN:creatinine ratios were measured (Fig. 4C). While albumin levels remained mostly constant, a spike in globulin levels and drop and albumin-globulin ratios were evident in virulent strain infected dogs independent of Sheila Smith or Morgan strain used for infection (Fig. 4D–F). Likewise, a transient increase in serum alkaline phosphatase activity was evident in dogs when receiving either one of the virulent strain infections on day 3 post-infection which returned to normal range thereafter (Fig. 4G).

### 2. Whole-cell antigen vaccinations prevented severe clinical disease and mortality following heterologous and homologous *R. rickettsii* strain infection challenges.

We then tested the immune protection in the canine infection model with vaccines prepared using whole cell inactivated antigens of the two virulent strains (Sheila Smith and Morgan) and the avirulent (Iowa) strain. Independent of the vaccine formulation prepared, all dogs from vaccinated groups were protected against virulent infection challenges with either one of the two virulent strains.

#### 2.1. Clinical signs and severe disease were absent for all vaccinees receiving virulent strain infections.

The above-described findings demonstrate that infection with the two virulent strains induced classical acute RMSF disease and resulted in 60% of animals developing morbid disease requiring euthanasia. We next evaluated the protective efficacy of WCAVs prepared from the avirulent (Iowa) strain as well as the virulent strains (Sheila Smith and Morgan). Five groups of dogs (n=5 per group of both sexes) received primary vaccination (on day zero) and booster vaccination (on day 21). Subsequently, all five groups were challenged with the two virulent strains on day 42 to assess vaccine-mediated protection. Dogs vaccinated with Iowa- or Morgan-derived antigen preparations were each subjected to heterologous challenge with either Sheila Smith or Morgan strains. As we previously reported homologous challenge protection for the Sheila Smith strain vaccine^40,41^, we only tested Sheila Smith strain-derived vaccine challenged with Morgan strain virulent infection (Fig 5.). All five vaccinated groups remained clinically stable and did not develop serious illness irrespective of virulent challenge strain used (Sheila Smith or Morgan) (Fig. 5A and B). These observations are also independent of whether the vaccine was prepared from the avirulent Iowa strain or from the virulent Morgan or Sheila Smith strains and similarly, independent of either heterologous or homologous strain infection challenges. All vaccinees exhibited markedly attenuated clinical signs throughout the 14-day acute phase, as per the clinical severity score assessment (Fig. 5C and D, Table S1). AUC analysis revealed significant reductions in cumulative disease burden in all vaccinated groups compared to unvaccinated controls receiving virulent infection challenges with either Sheila Smith or Morgan strains (Fig. 5E and F). All vaccinated dogs, independent of the vaccine formulation used, and following the virulent infection challenges, maintained normal body temperatures (Fig. 6A and B). A brief spike in body temperature was observed one day post administration of the booster vaccine for the dogs receiving either of the virulent strain antigen vaccines and resolved within a day. In contrast, unvaccinated infection controls developed fever beginning on day 2 post-challenge and persisting for several days as reported above in Fig 2. No weight loss or gain in the body weights was observed in vaccinees as compared to unvaccinated dogs receiving infection challenges with the virulent strains, which displayed steady weight losses (Fig. 6C and D).

#### 2.2. Hematological values remained unchanged for all vaccinated groups following infection challenges with either of the two virulent strains of *R. rickettsii*.

Hematological parameters, including RBC, monocytes, lymphocytes, eosinophils, and neutrophils, remained within normal limits across all vaccinated groups, whereas unvaccinated dogs challenged with virulent *R. rickettsii* exhibited significant reductions in these cell populations following challenge (Fig. 7). The vaccine-associated protective effect in preventing all cytopenias was also evident independent of either virulent or avirulent strain(s) vaccine preparation of antigens. Contrary to the notable changes in the blood chemistry values observed for the unvaccinated virulent *R. rickettsii* infected dogs, which included BUN, BUN:creatinine ratio, albumin:globulin ratio, and spike in the alkaline phosphatase levels, all vaccinees receiving virulent infection challenges had normal profiles (Fig. 8).

#### 2.3. *R. rickettsii* infection was reduced in all vaccinated dogs following virulent infection challenges.

The presence of *R. rickettsii* in the circulation and tissues was evaluated using two independent methods: 1) *in vitro* culture recovery to detect viable bacteria replicating in Vero cells, and 2) TaqMan probe-based real time PCR (qPCR) assay targeting the *sca4* gene. Blood samples collected on days 3, 7, 11 and 15 post-challenges were used to infect Vero cell monolayers. All five unvaccinated dogs receiving the Iowa strain infection tested negative for the culture recovery for the four time points assessed. Two dogs and one dog each from the Morgan-infected control groups tested positive on days 3 and 7, respectively (Table 1). Similarly, three dogs, two dogs and one dog each from the Sheila Smith strain-infected group were positive on days 3, 7, and 11, respectively (Table 1). Whereas none of the vaccinated dogs tested positive for the culture recoverable *R. rickettsii* for all four time points assessed, **r**egardless of the vaccine source (Table 1). Genomic DNAs extracted from blood samples collected at various time points following vaccination and virulent infection challenges, and from tissues collected during the terminal time points were assessed by qPCR (Fig. 9 and Table S2). While all dogs tested negative post vaccinations prior to infection challenge, both unvaccinated and vaccinated dogs receiving infections tested positives for some time points (mostly within the first 9 days) with significantly less positives observed in the vaccinated groups compared to unvaccinated groups (Fig. 9 and Table S2). Similar evaluation of tissue DNAs by qPCR at the terminal time points of the study yielded significantly more positives for groups of virulent infected control dogs (Fig. 9B and Table S3).

#### 2.4. Vasculitis marker, ICAM-1, expression was higher only in the unvaccinated controls receiving virulent infection challenges.

Vascular involvement is a hallmark for *R. rickettsii* infection^24,42^. We quantified plasma concentrations of Intercellular Adhesion Molecule-1 (ICAM-1), a marker of endothelial activation frequently associated with vasculitis (Fig. 10). Unvaccinated dogs receiving Sheila Smith infection produced the highest amount of ICAM-1 which peaked on day 7 post-infection and remained elevated through day 11, in contrast, Morgan and Iowa strain-infected dogs had less ICAM-1 expression (Fig. 10A). ICAM-1 levels were lower throughout infection assessment for the vaccinated dogs compared to the unvaccinated infection controls, although the data were only significant for the Sheila Smith-infection challenge groups (Fig. 10B and C).

#### 2.5. Histopathological analysis revealed minimal or no lesions in vaccinated dogs and in Iowa-infected dogs.

Histological examination of tissues collected at terminal time points revealed severe pathological changes exclusively in unvaccinated infection controls challenged with virulent strains. In contrast, minimal pathology was observed in dogs infected with the avirulent Iowa strain and in vaccinated animals following virulent strain challenge. In Iowa controls, lesion scores were the least for liver and lung, while all other organs had normal histology (Fig. 11 and Table 2A). Six dogs from virulent infection control groups exhibiting severe clinical signs and requiring euthanasia on days 11, 15 or 18 post-infection challenge and these dogs had severe pathology scores (Table 2A). These represent three animals each infected with Sheila Smith or Morgan strain (identified with gray highlighted text in Table 2A). These dogs had multisystemic lesions with the highest severity ranging between 3–5 for thymus, brain and testis (Table 2A). The predominant observations were that of having nonsuppurative inflammation which ranged from lymphocytic to granulomatous, composed mainly of lymphocytes, plasma cells, and histiocytes. Neutrophilic inflammation was also observed in testes and spleen tissues. Vasculitis was a prominent feature across multiple organs, having fibrinoid vasculitis observed within the brain and testes tissue sections. In lungs, inflammatory infiltrates were primarily perivascular, whereas in the liver, inflammation was typically multifocal and randomly distributed within the sinusoids. In one dog belonging to the Sheila Smith infection control group (Dog ID: SOT), the liver showed marked neutrophilic inflammation which was frequently localized around the central veins. Four dogs from the two virulent infection control groups survived, similarly all five dogs from the Iowa infection control group had only mild lesions, which were limited to low-grade inflammatory changes in isolated organs (Table 2 and Fig. S2). Contrary to these observations, and irrespective of vaccine antigens used, all vaccinees displayed mostly minimal lesion scores (Table 2B and C).

#### 2.6. All three formulations of WCAV elicited higher humoral immune response.

Independent of the vaccine preparation, WCAVs induced strong antigen-specific IgG responses (Fig. 12). The IgG response was higher when Iowa antigens were used to measure the response in the ELISA assays, whereas the antigens from both the virulent strains detected higher response only following booster vaccinations (Fig. 12 A–C). The IgG response remained similar for the controls following infection challenges with either of the virulent strains (Fig .12 A–C). IgG1 and IgG2 isotype antibodies were detected nearly equally in dogs receiving the vaccinations (Fig. 12 D–I), whereas the unvaccinated infection controls primarily had an IgG2 isotype antibody response.

#### 2.7. Vaccinations elicited robust antigen-specific CD4^+^ and CD8^+^ T cell responses which remained after virulent *R. rickettsii* infection challenges.

PBMC stimulation with whole cell antigens and the assessment of T cell proliferation revealed the vaccine-specific expansion of both CD4^+^ and CD8^+^ T cells in all dogs vaccinated with either Iowa, Sheila Smith or Morgan-WCAV (Fig. 13 A and B). CD4^+^ T cell proliferation was increased in Morgan- and Sheila Smith-vaccinated animals upon stimulation with homologous antigens, with significantly higher proliferation compared to unvaccinated dogs (*P* < 0.05) (Fig. 13 A). CD8^+^ T cell responses showed similar pattern, although at slightly lower levels (Fig. 13 B). On day 7 post-infection challenges, CD4+ T cells responses were maintained, whereas CD8+ T cells responses varied depending on the antigen used for stimulation and the vaccination group, indicating a heterogeneous cellular response (Fig. 13 C and D).

## DISCUSSION:

RMSF remains a high-impact disease in humans and dogs as it is responsible for causing high fatalities, particularly in younger children in the regions where the infection is highly prevalent, such as in parts of north, central and south American countries^1–3,6–8,43,44^. Currently, there is no licensed vaccine available to prevent the disease in either dogs or humans. Dogs are clinically relevant sentinels and play a critical epidemiological role for the disease thus, reinforcing our one health approach for developing an effective canine vaccine^45,46^. In this study, we utilized the canine infection challenge model for the RMSF to address a key translational gap: 1) whether a whole-cell antigen vaccine (WCAV) can protect against two genetically distinct virulent *R. rickettsii* strains; Sheila Smith and Morgan and 2), can a WCAV prepared from an avirulent strain abate the progression of disease against the two virulent infections challenges. Two major findings emerged from the current study. First, the Iowa strain, a known avirulent version of *R. rickettsii*, was found to be non-pathogenic in dogs as it caused a minimal clinical disease with 100% survival compared with the Morgan or Sheila Smith strain infections leading to 60% fatalities, thereby establishing a clear virulence gradient in a natural host. Secondly, all three inactivated whole-cell antigen vaccine formulations made from Iowa, Morgan, or Sheila Smith strain conferred strong immune protection against virulent heterologous infection challenges, preventing mortalities and markedly reducing clinical disease, systemic inflammation, pathogen burden, and tissue pathology. Together, these data support that whole-cell inactivated vaccination serves as a robust platform for cross-strain RMSF protection in a clinically relevant host.

The WCAV prepared using the avirulent Iowa strain can be a safer alternative for future application of the vaccine in respect to human use. An important contribution of this work is the confirmation that the Iowa strain infection causes very mild pathogenicity in dogs, extending observations previously restricted to small animal models^25,28,47^. Iowa strain has long been recognized as attenuated or avirulent in guinea pigs where it produces reduced fever, limited pathology, and survival even at doses lethal with virulent strains^25,28,47^. Our findings demonstrate that the attenuation phenotype is also observed in the physiologically relevant canine host, one that naturally develops RMSF following *R. rickettsii* infection with fatal outcomes if left untreated. Consistent with the prior data suggesting the weakened virulence, we demonstrated that Iowa strain-infected dogs exhibited minimal clinical signs, negligible endothelial activation (as reflected by ICAM-1), and only having mild localized histological changes. Thus, the dog model faithfully recapitulates Iowa attenuation observed in guinea pigs and supports the concept that the strain represents a biologically meaningful low virulence-associated strain that is ideally suitable for use as a safe whole cell antigen vaccine preparation sourcing and heterologous challenge benchmarking.

The central objective of the current study was to assess the WCAV protection against heterologous strain challenges. Inactivated WCAV formulations using three different sources of antigens prevented mortality and attenuated disease following virulent infection with both Sheila Smith and Morgan strains. Protection was observed regardless of whether the vaccine was prepared from the avirulent Iowa strain or either one of the two virulent strains, indicating broad protective efficacy of WCAVs. This finding is particularly important in light of the historical difficulty of developing effective RMSF vaccines^40^. Subunit vaccine approaches targeting major surface antigens such as OmpA or OmpB, Adr2 have shown immunogenicity but only partial or inconsistent protection in experimental small animal models, as well as in the canine host model^34,35,38–40^.

The whole-cell antigen vaccine is expected to provide a broader antigenic repertoire, increasing the probability of targeting conserved protective determinants and enabling effective cross-strain immunity. Notably, Iowa strain-derived WCAV performed similarly to vaccines prepared from virulent strain antigens, despite it being a non-pathogenic strain. Live infection of guinea pigs with Iowa strain confers protection against subsequent challenge with the virulent strain *R. rickettsii* Sheila Smith^28^. This study further supports our data that the WCAV prepared from Iowa is efficacious in conferring protection against the two virulent strains of *R. rickettsii* and is also likely that it extends protection against other untested virulent strains. Iowa strain-derived WCAV, therefore, is expected to serve as a safer vaccine option in avoiding any risks associated with live or replicating organisms.

Dogs infected with virulent strains reproduced hallmark clinical and clinicopathologic features of canine RMSF. The features included fever, transient weight loss, thrombocytopenia, lymphopenia, and biochemical perturbations such as the drop in BUN, BUN: creatinine ratio, albumin: globulin ratio, spike in alkaline phosphatase levels, as well as the higher expression of vascular damage marker (ICAM-1), and having gross lesions consistent with a severe form of RMSF and reflecting systemic inflammation, vascular dysfunction, and secondary renal and hepatic functional impairment. These manifestations have been well described in experimental and naturally occurring canine RMSF which closely mirrors severe form of human RMSF disease^16,17,40,41,48^. Importantly, WCAV reduced clinical scores across all assessed indicators of the disease severity. Vaccinated dogs also maintained nearly normal blood cell profiles and other biochemical markers. These convergent endpoints reinforce that the vaccine-mediated protection translates into biological suppression of the systemic disease processes driven by rickettsial endothelial infection.

We used two complementary pathogen readouts to demonstrate vaccine protection: recovery of viable bacteria in cell culture and PCR detection of *R. rickettsii* DNA in blood and tissues. The concordance of these approaches supports a strong conclusion that WCAV suppresses bacteremia and markedly limits tissue dissemination or damage. Disease severity in RMSF is tightly linked to vascular endothelial infection and vascular inflammation^24^. ICAM-1 is a well-established marker of vascular inflammation and vasculitis, and its elevation correlates with disease severity and vascular injury^49,50^. Thus, elevated ICAM-1 expression can allow endothelial activation and leukocyte recruitment in rickettsial infections^51^. In this study, virulent-strain infections alone showed pronounced ICAM-1 responses, whereas the vaccinated animals reduced it to near baseline. Histopathological findings paralleled these data: multisystemic lesions, including vasculitis, were observed with virulent-strain infections, while vaccinated dogs independent of the antigen source from avirulent or virulent strains displayed minimal or no pathology. The alignment of circulating vascular markers with tissue-level improvements provides a coherent mechanistic link between vaccination and disease suppression.

Adaptive immunity is essential for protection against the disease. All WCAV formulations elicited robust antigen-reactive IgG responses across homologous and heterologous ELISA antigens, indicating broad cross-reactivity. Following infection challenges, vaccinated dogs showed only modest antibody boosting, consistent with rapid pathogen control and limited antigenic stimulation. Antibodies produced by B cells contribute to host protection through Fc-dependent effector mechanisms that promote pathogen clearance and limit host cell infection^52^. Prior studies on other *Rickettsia* species demonstrate that vaccination-induced serum IgG antibodies correlate with protection against rickettsial infection and possess direct neutralizing effects on the pathogens^52,53^. It is highly likely that the WCAV formulations we tested in the current study elicit IgG antibodies with expansion of Th1 and Th2 type-specific IgG1 and IgG2 subclasses which may critically contribute to neutralization of *R. rickettsii*. This hypothesis remains to be tested.

Based on prior demonstration of broader and stronger immune protection exerted by the WCAV with Quil A^41^, in the current study, we used the same adjuvant in all vaccine formulations used. Quil A-adjuvanted whole cell vaccines are also known to favor cell-mediated immune responses, including CD8^+^ T cell activation^54^. We recently tested Montanide, Alhydrogel and Quil A adjuvants and demonstrated that WCAV protect dogs independent of the adjuvants used, although we observed a difference among the three adjuvants with Quil A having elicited the highest IgG responses accompanied by a significant increase in both CD4^+^ and CD8^+^ T-cell numbers^41^. In this study, vaccinated dogs exhibited similar antigen-responsive CD4^+^ and CD8^+^ T cell proliferation before and after challenge, consistent with primed cellular immunity^42,55^. Findings in the current study were similar to our previous published work demonstrating the expansion of IFN-γ–producing CD4^+^ and CD8^+^ T cells in vaccinated dogs and supporting a role for Th1-associated cellular immunity in protection against *R. rickettsii*^41^. Although IgG subclass patterns alone cannot definitively define Th1/Th2 polarization in dogs, the combination of IgG responses and antigen-specific T cell proliferation is consistent with a protective immune profile involving both humoral and cellular immunity. The most defensible interpretation is that effective protection reflects synergy between cross-reactive antibodies and rapid cellular recall responses that together suppress bacteremia and dissemination. Interestingly, the avirulent Iowa strain-infected control dogs developed very minimal to no clinical signs and survived without the need of any vaccination. This outcome likely reflects the intrinsically attenuated nature of the Iowa strain, which imposes a reduced requirement for adaptive immune engagement. Early control of bacterial replication may therefore be achieved predominantly through innate immune mechanisms, limiting antigen burden and disease progression. In this context, antibody responses may arise secondary to infection but are not the primary determinant of protection. By contrast, infection with virulent strains likely exceeds the capacity of early innate defenses, necessitating a more robust and timely adaptive response required to prevent severe disease development and the associated mortality. Consistent with this interpretation, previous studies have shown that early innate immune responses contribute critically to the control of rickettsial replication before the development of adaptive immunity^56,57^.

Dogs serve as both sentinel hosts and amplifiers of infection and pose a higher human RMSF risk, particularly in regions where *Rhipicephalus sanguineus* drives peridomestic transmission^45,46,58,59^. Effective vaccination of dogs could reduce the burden of RMSF in both the companion animals and humans in proximity by limiting canine RMSF disease and reducing bacteremia. This study provides strong evidence that inactivated whole-cell antigen vaccines confer robust cross-strain protection against virulent *R. rickettsii* infection challenges in dogs. We demonstrated that the Iowa strain is attenuated with presenting a very mild disease in dogs and that inactivated antigens derived from this strain confer protection against heterologous virulent strain challenges. The data support a rational path toward the development of a safe and efficacious vaccine preventing fatal RMSF. Such a vaccine could reduce disease risk in the companion animal and, in turn, have an important public health impact by lowering the risk of human RMSF cases and the associated morbidity and mortality.

## METHODS:

### Propagation of *R. rickettsii* in Vero cells for preparation of stocks.

*R. rickettsii* strains (Sheila Smith, Morgan, and Iowa) were propagated in Vero cells (African green monkey kidney; clone E6, ATCC CRL-1586) as previously described^40,41^. Briefly, confluent Vero monolayers grown in Dulbecco’s modified Eagle medium supplemented with 2% fetal bovine serum and 2 mM L-glutamine were infected at a multiplicity of infection (MOI) of one and incubated at 34°C with 5% CO_2_ until approximately 50% of the monolayer showed infection-associated cytopathic effects. Lysates from infected cultures were subjected to differential centrifugation, and pelleted *R. rickettsii* were resuspended in K-36 buffer (0.1 M potassium chloride, 0.015 M sodium chloride, 0.05 M potassium phosphate buffer; pH 7.0). Viable *R. rickettsii* were quantified by plaque titration and qPCR.

### Whole-cell antigen preparation and vaccine formulations.

Whole cell antigen (WCA) lysates were prepared as described previously^40,41^. Briefly, cultured organisms from Vero cells were incubated in a 56°C water bath for 30 min, with mixing once every 10 min. The protein concentration was estimated according to the BCA (Bicinchoninic Acid) method (ThermoFisher scientific, Waltham, MA). In this study we used and compared three different vaccine preparations for use as vaccines with Quil A as the adjuvant (InvivoGen, San Diego, CA): Sheila Smith WCA-V, Morgan WCA-V and Iowa WCA-V. Vaccines were prepared by mixing with 70 μg per ml of inactivated *R. rickettsii* whole-cell antigen (Sheila Smith, Morgan or Iowa strains) diluted in PBS containing Quil A at final concentration of 250 μg.

### Experimental infections in dogs.

All experiments involving dogs were conducted in compliance with the Public Health Service Policy on the Humane Care and Use of Laboratory Animals and the U.S. Department of Agriculture (USDA) Animal Welfare Act and regulations, with approval from the University of Missouri Institutional Animal Care and Use Committee. Purebred beagle dogs (6 months old, both sexes) were obtained from a USDA Class A vendor and housed in climate-controlled indoor facilities at the University of Missouri. Dogs were provided with commercial dry food and water *ad libitum* and maintained with adequate space to allow regular exercise and free movement. Three groups of dogs received a primary and booster vaccination on days 0 and 21: 1 mL of Sheila Smith WCA-V (n = 5), 1 mL of Morgan WCA-V (n = 10), or 1 mL of Iowa WCA-V (n = 10). Control dogs (n = 15) received Quil A alone. The vaccines were administered subcutaneously. On day 42, all dogs were intravenously inoculated with 1×10^6^ *R. rickettsii* organisms in 1 mL PBS. Control mock vaccinated dogs, five per group, were challenged with one of the three different strains; Sheila Smith, Morgan or Iowa. All dogs received diphenhydramine syrup (4 mg/kg body weight) 30 min prior to infection challenge to reduce the risk of anaphylactic reactions^60^. All dogs were monitored daily for clinical, behavioral, and general health parameters, and hematological changes were assessed twice weekly by complete blood count analysis using the ProCyte DX (IDEXX, Westbrook, ME, USA) as previously described^41^. Body weight and body temperature were recorded according to the same procedures, with weights obtained weekly and temperatures measured twice weekly during vaccination and daily after infection challenge at consistent times of day. Blood samples were collected from the cephalic veins into EDTA tubes every 2–3 days starting on day 0 and continuing until the end of the study. Due to the development of severe clinical signs, six control dogs were euthanized on days 11, 15, and 18 post-infection (three each representing infections received with Sheila Smith or Morgan strain). At study completion, all remaining animals were humanely euthanized in accordance with the recommendations of the American Veterinary Medical Association (AVMA) Panel on Euthanasia, using a commercial pentobarbital-based euthanasia solution (Fatal-Plus; 0.22 mL/kg, equivalent to 86 mg/kg, administered intravenously).

### Clinical scoring system (0–3 scale): Clinical score and AUC calculation.

Clinical signs were monitored daily from day 0 (infection) until the study end day (day 79–80 depending on the challenge group). For quantitative comparison of disease kinetics, we focused on the acute phase of infection (day 0 to day 14), during which clinical signs in unvaccinated control dogs consistently peaked. For each dog, clinical disease was scored daily using a standardized ordinal scale from 0 to 3, where 0 = normal, 1 = mild signs, 2 = moderate signs, and 3 = severe signs requiring close monitoring or humane endpoints. Appetite, demeanor (BAR/QAR), mucous membrane color, hydration status, weakness, and other systemic signs were included in this composite score. To quantify cumulative disease burden for each animal, we calculated the area under the curve (AUC) of the daily clinical severity scores from day 0 to day 14 using the trapezoidal rule^61^:

$$AUC_{dog}=\sum_{i=1}^{n=1}\left(\frac{s_{i}+s_{i+1}}{2}\right)\left(t_{i+1}-t_{i}\right)$$

where *s*_*i*_ is the clinical severity score of that dog at time point *t*_*i*_, *t*_*i*_ is the day post infection (in days), and n is the total number of time points within the 0–14-day window. AUC values are expressed in arbitrary units (score × days), with higher AUCs reflecting more severe and/or prolonged clinical disease. while a lower AUC indicates protection. AUC values for animals euthanized before day 14 were calculated using the observed clinical scores available until the day of euthanasia and therefore represent cumulative disease burden up to the humane endpoint. Group data (e.g., for AUC bar graphs) represent the mean ± SEM of individual dog AUCs within each group.

### Culture recovery.

Blood samples collected in EDTA on days 3, 7, 11, and 15 post-infection were used for culture recovery. Plasma was removed from 4 mL of whole blood, and the buffy coat was transferred into a sterile 15-mL tube containing 5 mL of red blood cell lysis buffer (155 mM NH_4_Cl, 10 mM KHCO_3_, 0.1 mM EDTA). Samples were gently mixed until red blood cell lysis was complete, then centrifuged at 300 × g for 5 min, and supernatants discarded. The resulting buffy coat pellet was resuspended in 200 μL of sterile 1× PBS. A 100 μL aliquot of each suspension was added to individual wells of a 12-well culture plate containing an ~80% confluent Vero cell monolayer. Plates were incubated at 37°C in a CO_2_ incubator, and cultures were monitored for *R. rickettsii* growth following the procedures described above. To confirm infection, cultures were transferred to glass slides, fixed, and stained using the Hema3 protocol^60^. Cultures were examined for up to 30 days to detect the presence of *R. rickettsii*.

### Rickettsial DNA assessed by real-time TaqMan probe-based quantitative PCR (qPCR).

Whole-blood samples were collected aseptically into 4-mL EDTA vacutainer tubes (Corning Inc., Lowell, MA). For real-time PCR, genomic DNA was extracted using the DNeasy Blood and Tissue Kit (Qiagen, Valencia, CA) following the manufacturer’s instructions, and samples were stored at −20°C until analysis. Detection of *R. rickettsii* DNA was performed using a TaqMan probe-based qPCR assay targeting the sequence coding for the *sca4* gene, with primers and probe as described in Stoll *et al*.^62^. Amplification was conducted on a QuantStudio 3 real-time PCR system (Applied Biosystems, Waltham, MA) using the following cycling conditions: 94°C for 2 min, followed by 40 cycles of 94°C for 15 s and 61°C for 50–60 s. Reactions were performed in 25 μL volumes containing 2 μL DNA template, 1.5 mM MgCl_2_, 0.2 mM each dNTP, 0.5 μM forward and reverse primers, 0.25 μM TaqMan probe, and 1.25 U Platinum Taq DNA Polymerase (Invitrogen, Waltham, MA). Data were analyzed using Applied Biosystems Design & Analysis Software v2.7.0. A sample was considered positive only when clear exponential amplification was observed and all assay controls performed as expected, including the absence of amplification in no-template controls. Samples with a Ct value ≥38 were considered negative.

### Quantification of Intracellular adhesion Molecule-1 (ICAM-1).

ICAM-1 levels were analyzed for different time points by using commercial ELISA kits (Cusabio Dog intercellular adhesion molecule 1, ICAM-1/CD54 ELISA Kit). The analyses were performed according to the instructions from the manufacturer.

### Histopathology analysis.

Histopathological assessments were conducted as previously described^41^. Briefly, gross and microscopic tissue sampling was performed by a board-certified veterinary pathologist (DYK) blinded to vaccination status. Tissues collected from all dogs including lung, liver, thymus, kidney, spleen, heart, lymph nodes, bone marrow, testicle or mammary gland, and brain, were fixed in 10% neutral-buffered formalin, routinely processed, sectioned at 4 μm, and stained with hematoxylin and eosin. Lesion severity was scored using a semi-quantitative scale (1 = mild; 2 = mild–moderate; 3 = moderate; 4 = moderate–severe; 5 = severe) and classified as multifocal or diffuse. Predominant inflammatory cell types within affected areas were recorded as lymphocytic (L), neutrophilic (N), plasmacytic (P), or histiocytic (H).

### Humoral immune response.

ELISA analysis was performed to measure IgG response using inactivated whole-cell *R. rickettsii* antigens. Peripheral blood was collected in EDTA tubes, and plasma was obtained by standard centrifugation procedures. For the indirect ELISA (iELISA), 96-well Immulon^™^ 2HB plates (Thermo Fisher Scientific, Waltham, MA) were coated overnight at 4°C with 100 μL per well of inactivated *R. rickettsii* antigens (2 μg/mL) diluted in 1X carbonate–bicarbonate coating buffer (pH 9.6). Plates were blocked with 200 μL of blocking buffer (1% BSA in 1× PBS) for 1 h at 37°C. Plasma samples were diluted 1:200 in blocking buffer, and 100 μL of each sample was added in triplicate to the coated wells. After a 1h incubation at 37°C, plates were washed three times with a wash buffer (0.05% Tween 20 in 1XPBS). Secondary peroxidase-conjugated antibodies anti-canine total IgG (1:50,000; PA1–29738, ThermoFisher), IgG1 (1:10,000; AHP947P, Bio-Rad), or IgG2 (1:5,000; AHP948P, Bio-Rad), were added at 100 μL per well and incubated for 45 min at 37°C. Plates were washed three times, and 100 μL of TMB substrate was added and allowed to develop for 5 min at room temperature. The reaction was stopped with 50 μL of 1 M phosphoric acid, and absorbance was read at 450 nm using a Tecan Infinite microplate reader (Tecan Group Ltd., Männedorf Switzerland).

### Isolation of PBMCs and assessment of T cell expansion following *R. rickettsii* antigen stimulation.

PBMC isolation and T cell expansion assays were performed as previously described^63^. Briefly, 8–10 mL of peripheral blood samples were collected from each animal into sodium citrate CPT tubes (BD Biosciences, San Jose, CA, USA) at defined time points after WCA-V vaccinations and after challenge with the virulent Morgan and Sheila Smith strains of *R. rickettsii*. Tubes were centrifuged according to the manufacturer’s instructions, and the samples were shipped overnight to Iowa State University for analysis. Isolated PBMCs were washed twice in complete RPMI medium, counted, and resuspended to a final concentration of 4 × 10^6^ PBMCs/mL.

### Flow cytometry.

PBMCs were cultured for 5 days at 37°C with 4×10^5^ cells /well in 96-well plates and were stimulated with 10 μg/mL host cell-free Iowa, Morgan or Sheila Smith *R. rickettsii* whole-cell that was grown in Vero cells. As a positive control, cells were stimulated with 5 μg/mL Concanavalin A (Sigma-Aldrich). Following stimulation, cells were surface stained with mouse anti-canine CD3-FITC (clone CA17.2112), CD4-PECy7 (clone YKIX302.9), and CD8-(YCATE55.9), obtained from BioRad Laboratories (Hercules, CA, USA). PBMCs were resuspended in FACS buffer (0.1% NaN3, 10% fetal calf serum, and PBS) and incubated with primary antibodies at 10 μg/mL for 20 min at 4°C. Cells were then fixed using BD FACS Lysis buffer (BD Biosciences, San Jose, CA, USA). Flow cytometry data were acquired on a BD FACSCanto flow cytometer and analyzed with FlowJo software Version 10.10 (BD Biosciences, San Jose, CA, USA).

### Statistics.

Statistical Analyses Survival was assessed using Kaplan-Meier survival curves and comparisons were made using log-rank tests. The p-values for the specific paired comparisons from the linear regression (adjusted for Tukey multiple comparisons test). AUC values were calculated for each individual animal, and group means ± SEM were reported. Statistical comparisons between groups were performed using one-way ANOVA followed by Tukey’s multiple comparisons test. For Complete Blood Count and biochemical tests, statistical comparisons between groups were obtained by using two-way ANOVA followed by Dunnett’s multiple comparisons tests.

## Supplementary Files

This is a list of supplementary files associated with this preprint. Click to download.

- Table1.docx
- Table2.docx
- SupplementaryFiguresandTables.pdf

## Figures and Tables

**Figure 1.**
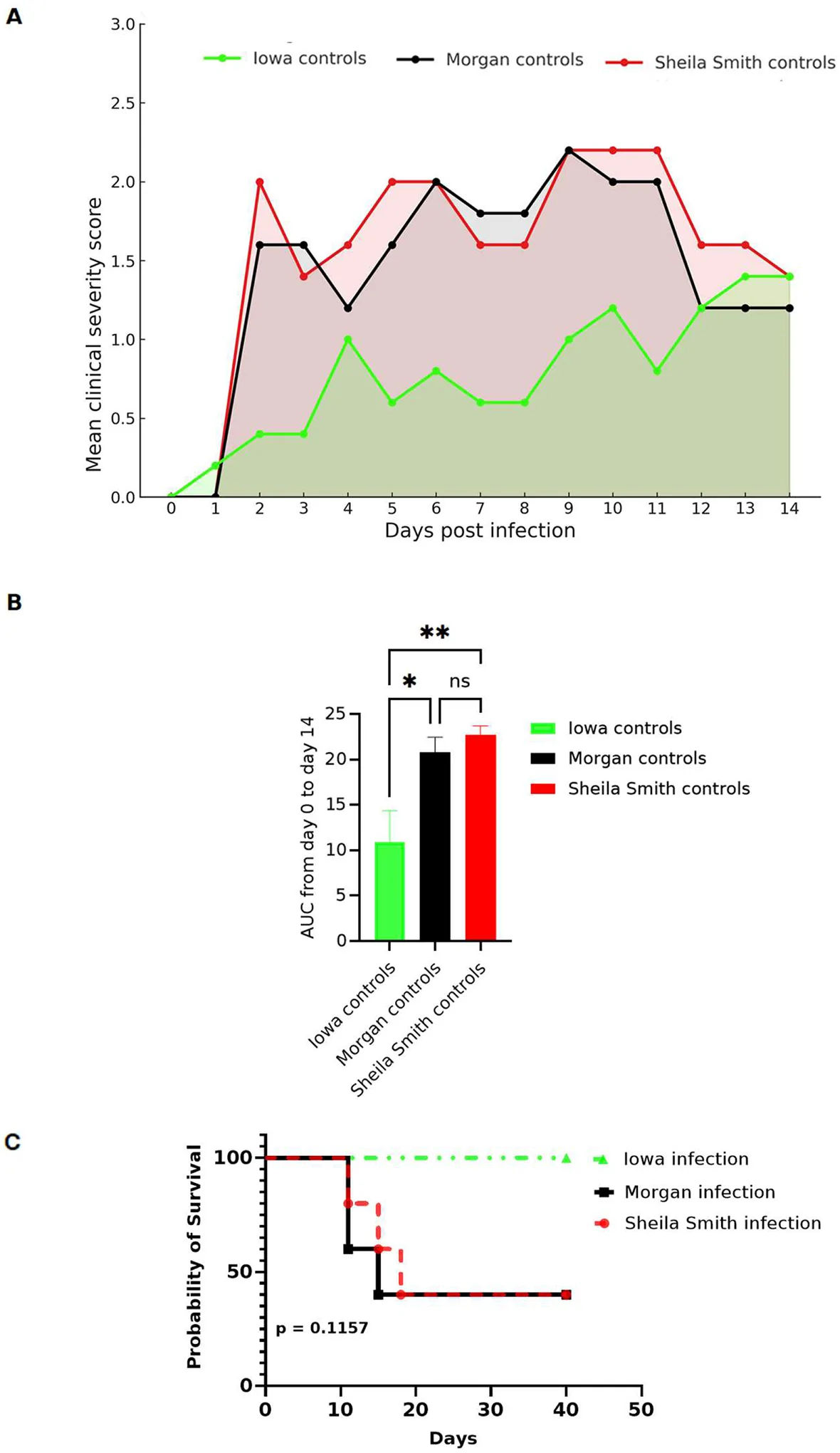
Clinical severity and survival of unvaccinated dogs infected with *R. rickettsii* Morgan, Sheila Smith, or Iowa strains. **(A)** Mean clinical severity scores (0–3 ordinal scale) recorded from day 0 to day 14 post-infection. Dogs infected with the Morgan and Sheila Smith strains had markedly higher and sustained clinical scores compared with the Iowa strain infected group, which exhibited only mild and transient signs. Colored shaded areas represent the corresponding AUC (Area Under the Curve) contribution for each strain (green, Iowa; black, Morgan; red, Sheila Smith). **(B)** AUC between day 0 to 14 to define clinical severity scores. Both Morgan- and Sheila Smith–infected dogs displayed significantly higher cumulative disease burden compared to Iowa-infected dogs (*p < 0.025; **p < 0.009; ns = not significant; one-way ANOVA with Tukey’s multiple comparisons test). Bars show mean ± SEM. **(C)** Kaplan–Meier survival curves of unvaccinated dogs. High mortality occurred in dogs infected with Morgan or Sheila Smith strains, while all Iowa-infected dogs survived to the study endpoint. Survival differences were assessed using the log-rank (Mantel-Cox) test - p = 0.112.

**Figure 2.**
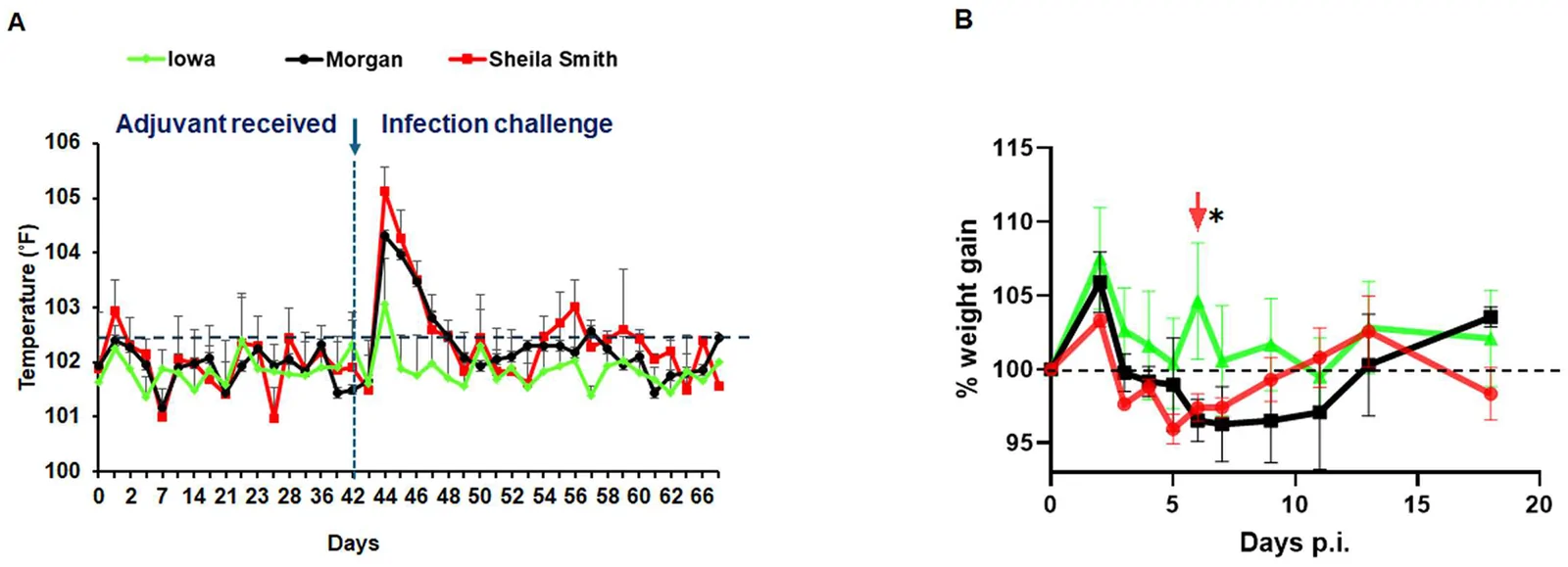
Temperature and body weight dynamics in unvaccinated control dogs challenged with three *R. rickettsii* strains. **(A)** Rectal temperature (mean ± SEM) monitored from day 0 to day 66. Dogs received adjuvant on day 0 and a booster on day 21, with no fever detected during this period. Following infection challenge on day 42, both Sheila Smith- and Morgan-infected dogs developed febrile response with temperature peaked to 105°F and 104.3°F respectively. Iowa controls remained normothermic. **(B)** Body weight changes (mean ± SEM) monitored from day o to day 20 post-infection (day 0 corresponds to the infection challenge day; i.e. day 42 of the study). Weight remained stable during the adjuvant-only phase (data not shown). After infection, Sheila Smith (red line) and Morgan (black line)-infected controls exhibited transient weight loss, whereas Iowa-infected dogs (green line) maintained stable body weight. Statistical comparisons were performed between groups using a two-way ANOVA with Tukey’s multiple comparison tests.

**Figure 3.**
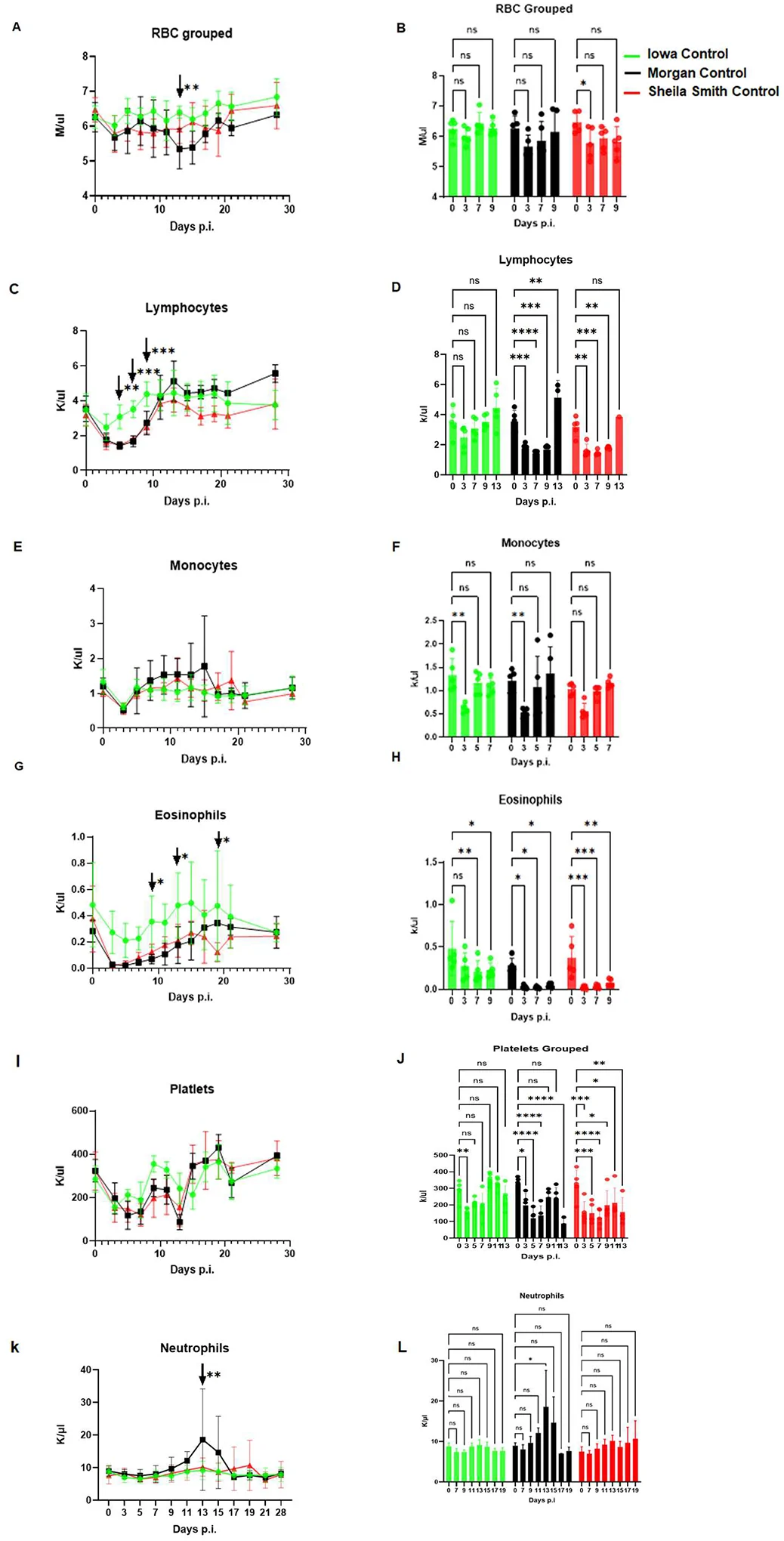
Hematological responses in unvaccinated control dogs following infection with three *R. rickettsii* strains. **(A–B)** Red blood cells (RBC). Mean RBC counts (± SEM) and grouped individual values across days post-infection. Sheila Smith–infected dogs showed a modest decrease at day 3 p.i., whereas Morgan-infected controls exhibited a transient reduction. Iowa controls remained stable. **(C–D)** Lymphocytes. Both Sheila Smith– and Morgan-infected dogs developed marked lymphopenia between days 3 and 9 p.i., followed by recovery toward baseline by days 13–15. Iowa controls maintained stable lymphocyte values throughout. **(E–F)** Monocytes. Monocyte counts showed early variability, with Iowa dogs displaying slightly higher values at early timepoints, whereas Sheila Smith and Morgan groups showed reduced and more variable counts. **(G–H)** Eosinophils. Eosinophil numbers decreased significantly in Sheila Smith– and Morgan-infected dogs at days 3 and 7 p.i., while Iowa controls maintained higher eosinophil levels across the same period. **(I–J)** Platelets. Sheila Smith infection induced transient thrombocytopenia beginning at day 3 p.i. and persisting through day 9. Morgan controls showed an intermediate pattern with milder decreases, while Iowa infection group maintained platelet counts within normal range. (**K-L**) Neutrophils. Circulating neutrophils in all infected dogs and for all time points were mostly stable except for the Morgan strain infected dogs on day 13. Statistical comparisons were performed between groups using a two-way ANOVA test followed by Dunnett’s multiple comparisons tests.

**Figure 4.**
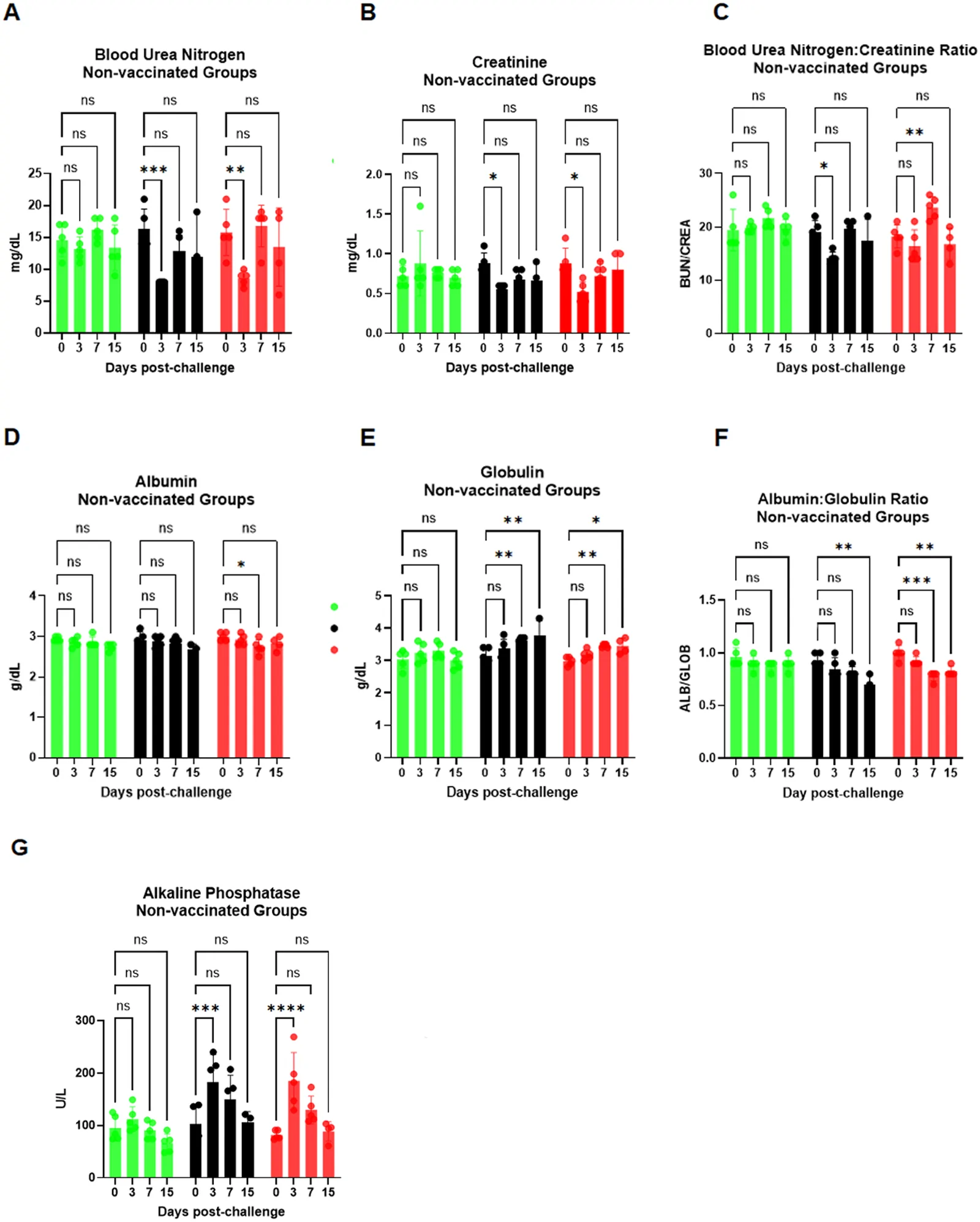
Biochemical parameters in unvaccinated control dogs following infection with three *R. rickettsii* strains. **A)** Blood urea nitrogen (BUN). **B)** Creatinine. **C)** BUN:creatinine ratio. **D)** Albumin, **E)** Globulin, **F)** albumin:globulin ratios, **G)** Alkaline phosphatase (ALP). Significant changes in these blood chemistry parameters following infection challenges were evident mostly for the virulent Sheila Smith– and Morgan-infected dogs between days 3–15 post infection challenges but not in Iowa-infected group. Statistical differences reflecting comparisons between groups were performed using a two-way ANOVA test followed by Dunnett’s multiple comparisons test.

**Figure 5.**
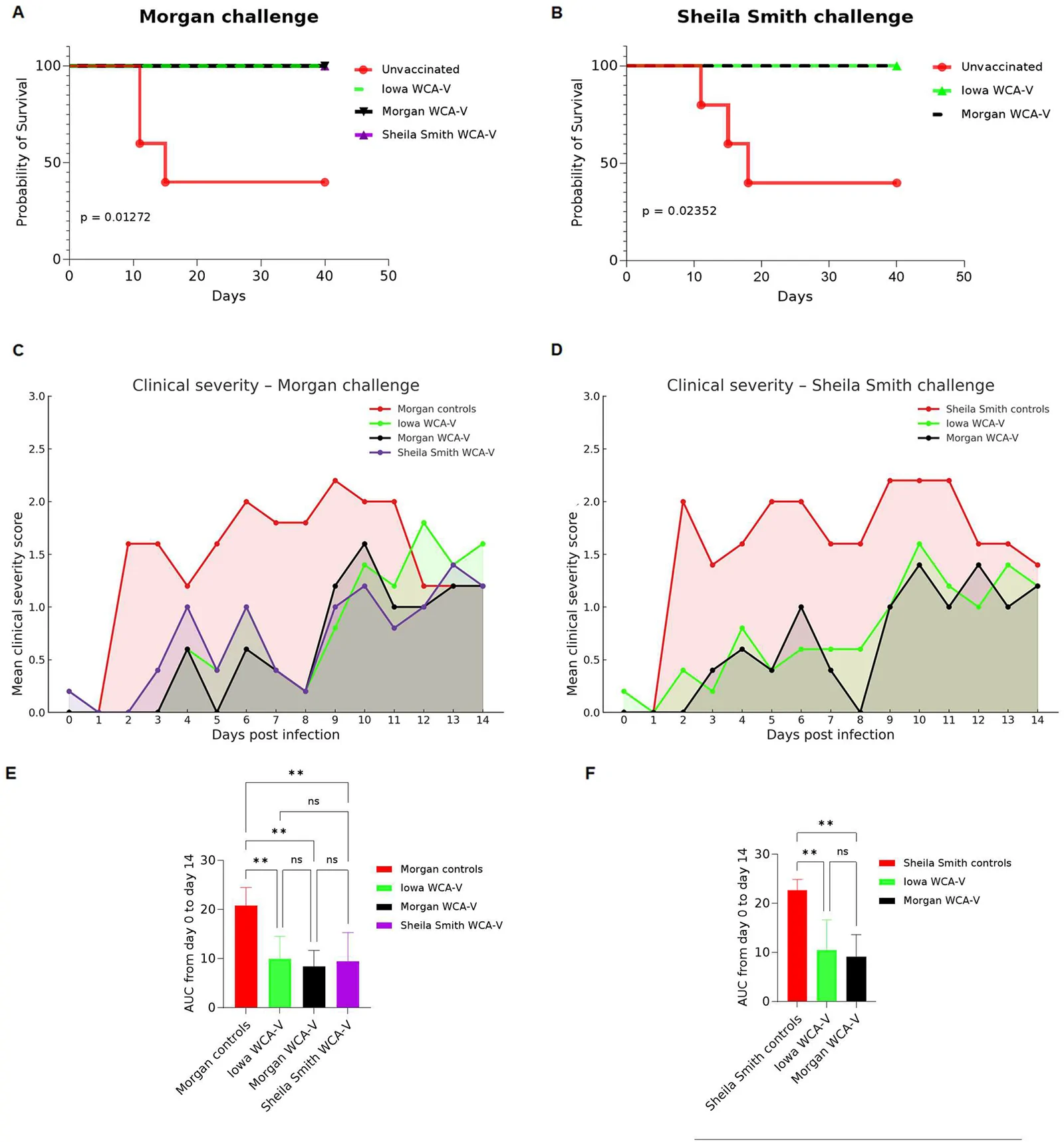
Protective efficacy of whole-cell antigen vaccines (WCAV) against homologous and heterologous *R. rickettsii* challenge in dogs. The left panels (**A, C** and **E**) show the results of the Morgan challenge, whereas the right panels (**B, D** and **F**) show the results of the Sheila Smith challenge. **A** and **B)** Kaplan–Meier survival curves of unvaccinated controls (red) and WCA-vaccinated groups with Iowa WCAV (green), Morgan WCAV (black), and Sheila Smith WCAV (purple), following Morgan challenge **(A)** or Sheila Smith challenge **(B).** Vaccination completely prevented mortality in dogs challenged with either the Morgan or Sheila Smith strains, in contrast to unvaccinated controls, which exhibited significant lethality (Morgan challenge: *p* = 0.01272; Sheila Smith challenge: *p* = 0.02352). **(C** and **D)** Mean clinical severity scores from day 0 to day 14 post-infection. Disease severity was markedly reduced in vaccinated dogs compared to unvaccinated controls. **(E** and **F)** Area under the curve (AUC; day 0–14) analysis of clinical severity scores. All vaccinated groups exhibited significantly lower AUC values compared to Morgan and Sheila Smith control groups, whereas no significant differences were observed among vaccinated groups. Shaded areas in severity plots represent SEM. Statistical analyses were performed using one-way ANOVA with appropriate post-hoc tests; ns = not significant, *p < 0.05, **p < 0.01, ***p < 0.001.

**Figure 6.**
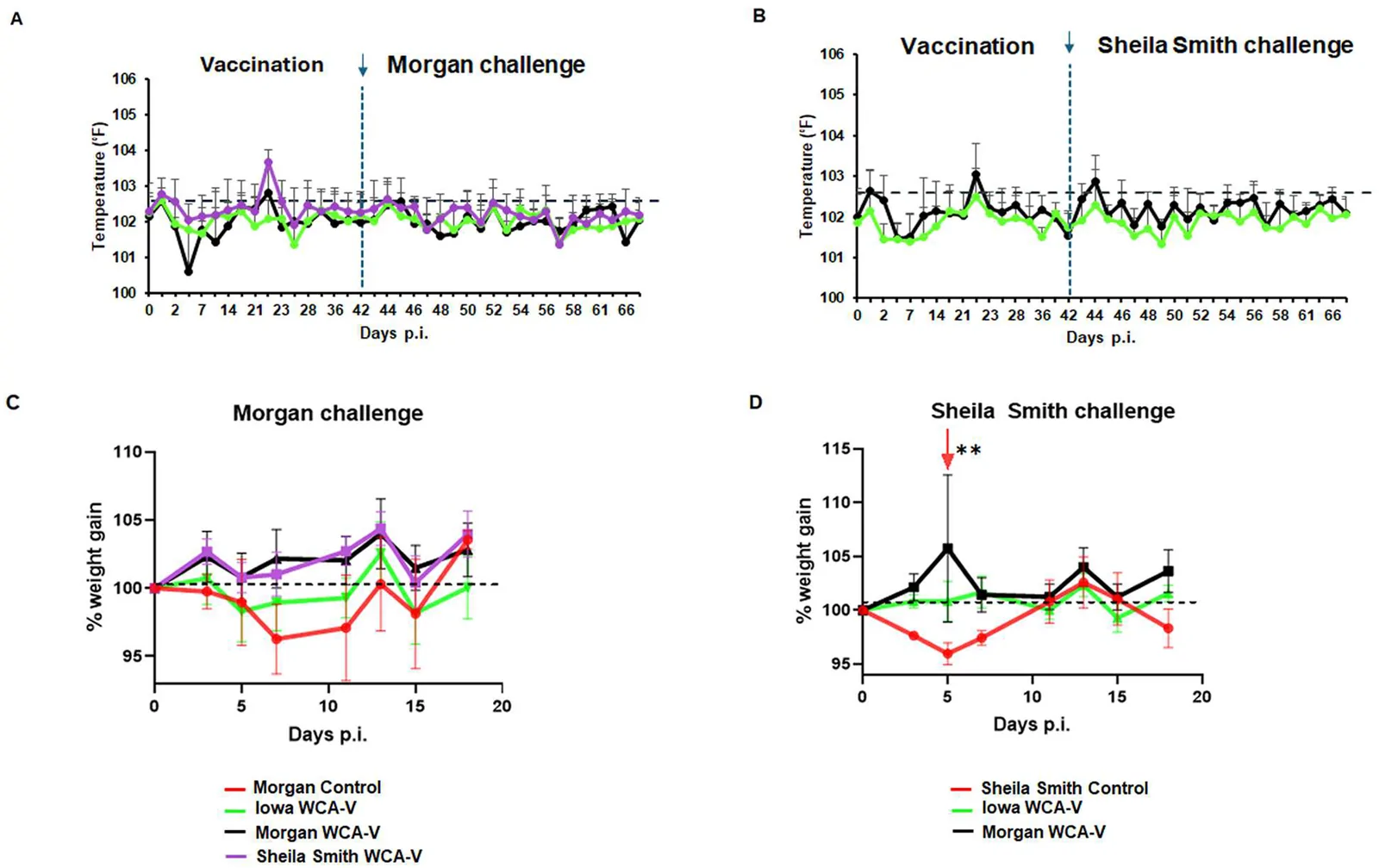
Temperature and weight dynamics following vaccination and challenge with R. rickettsii. Left panels show Morgan challenge (**A** and **C**) and right panels Sheila Smith challenge (**B** and **D**) for temperature and weight changes, respectively. Longitudinal rectal temperatures recorded from day 0 to day 66, encompassing the vaccination period (day 0 = first adjuvant/WCA administration; day 21 = booster) and subsequent Morgan (**A**) or Sheila Smith (**B**) infection challenges on day 42. All vaccine groups (Iowa WCA-V, Morgan WCA-V, Sheila Smith WCA-V), independent of the vaccine formulation used, maintained normal body temperatures after virulent infection challenges. A brief spike in body temperature was observed for one day after the booster vaccine for the dogs received Sheila Smith strain antigen vaccine. In contrast Morgan and Sheila Smith controls showed more pronounced febrile peaks. Morgan infection controls exhibited early weight loss following infection challenge, whereas vaccinated dogs largely maintained or gained weight throughout the acute phase (**C**); Sheila Smith infection controls showed significant early weight loss (notably at day 5), whereas both vaccine groups maintained stable weight or demonstrated modest gains **(D)**. Temperature and weight data are presented as mean ± SEM. Statistical comparisons were performed using repeated-measures analyses with appropriate post-hoc tests; ns = not significant, *p < 0.05, **p < 0.01.

**Figure 7.**
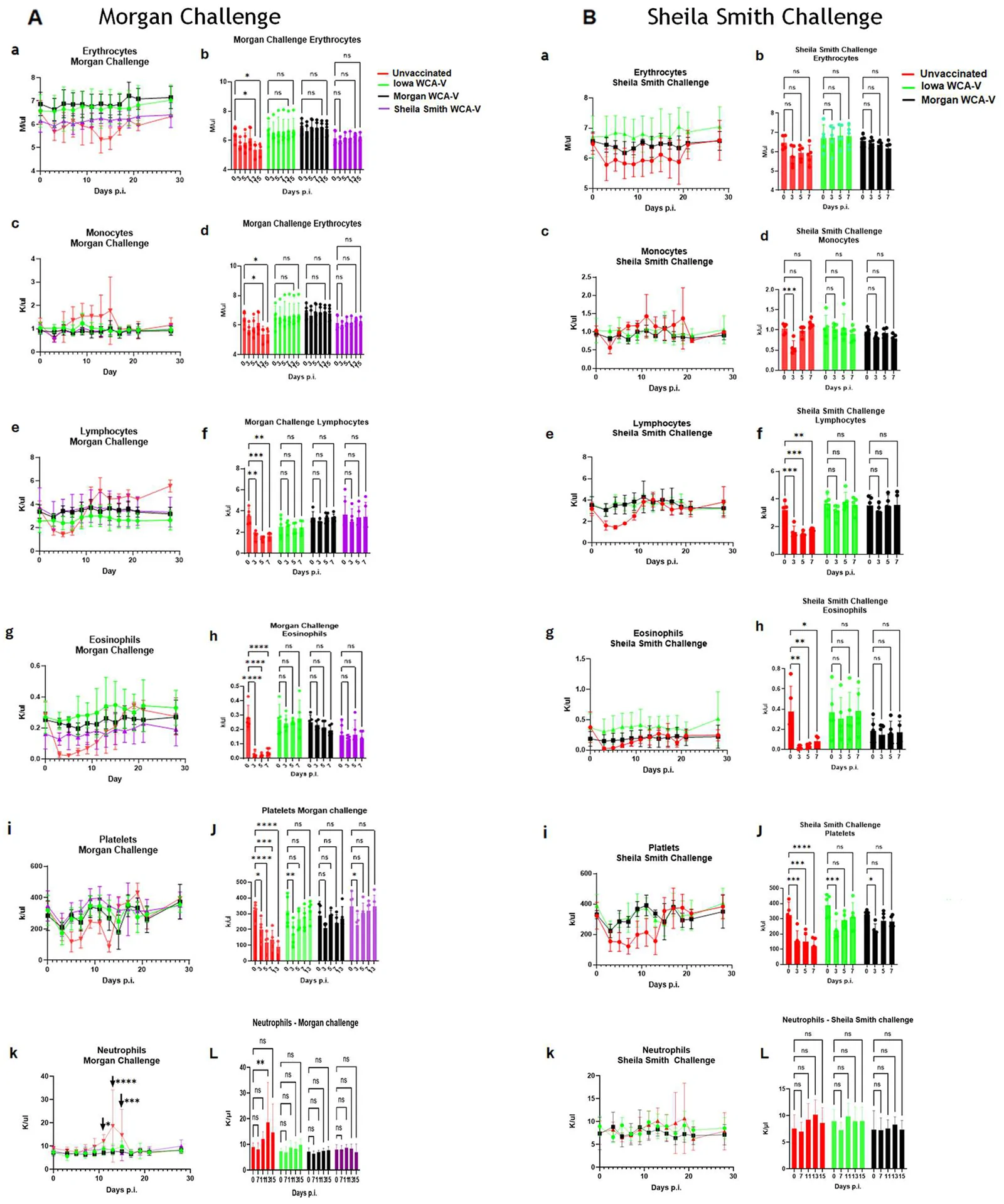
Hematological profiles in vaccinated dogs following Morgan or Sheila Smith challenge. Morgan infection challenge groups (left panels depicted under **A**) and Sheila Smith infection challenge groups (right panels depicted under **B**). Vaccinated animals challenged with either with the Morgan or Sheila Smith strain maintained overall stable hematological (blood cell) parameters compared with unvaccinated controls. Significant drops in all blood cells except for neutrophils were evident only in the unvaccinated infection controls with either one of the two virulent strain infections. Neutrophilia is more pronounced following Morgan strain infection controls and a tread of similar increase in neutrophils was observed in dogs following Sheila Smith infection; a significant difference in the neutrophilia was observed in the dogs in this group which developed the severe form of the disease (Fig. S1). Statistical comparisons were performed between groups using a two-way ANOVA test followed by Turkey’s multiple comparisons tests.

**Figure 8.**
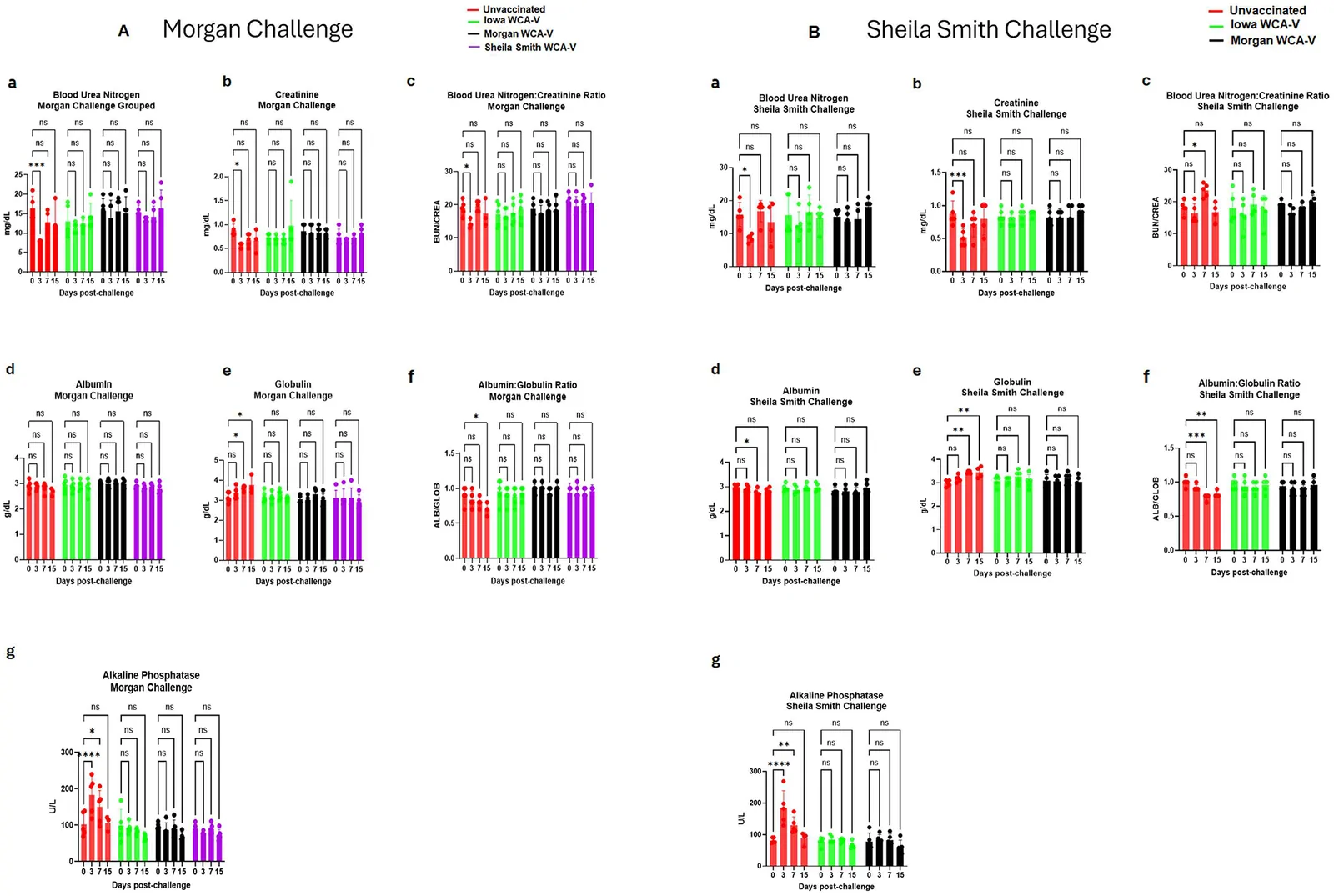
Serum biochemical parameters in vaccinated and unvaccinated dogs following *R. rickettsii* challenge. Panels **A** and **B** represent Morgan and Sheila Smith infection challenge groups, respectively and compared for groups receiving only infections or infections following vaccinations: blood urea nitrogen (a), creatinine (b), BUN/creatinine ratio (c), albumin (d), globulin (e), albumin:globulin ratio (f), and alkaline phosphatase (g) measured on days 0, 3, 7, and 15 post-challenge. (Iowa WCAV, green; Morgan WCAV, black; and Sheila Smith WCAV, purple, and unvaccinated controls identified in red color bars. Values represent mean ± SEM. Vaccinated dogs maintained stable biochemical profiles across all parameters compared to unvaccinated controls, which showed mild alterations following infection. Statistical comparisons between groups at each time point were performed using two-way ANOVA with Tukey’s post-hoc test.

**Figure 9.**
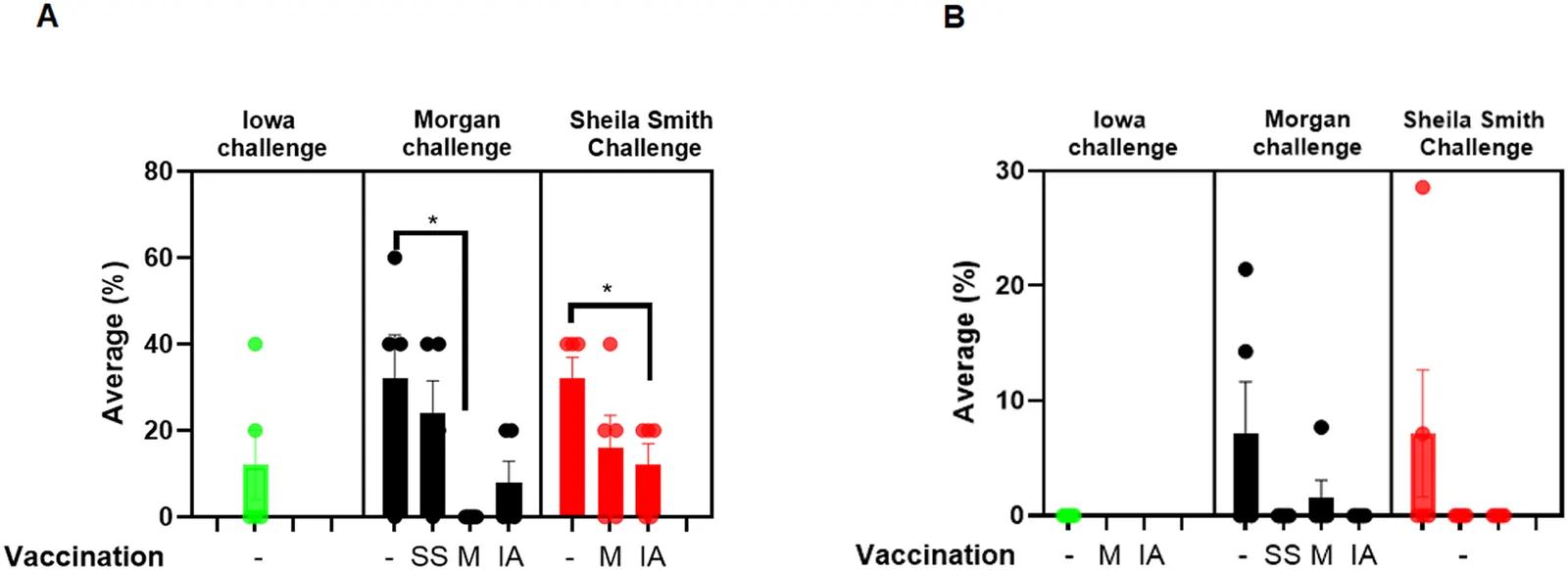
WCA vaccinations markedly reduce *R. rickettsii* bacterial burden in blood and tissues. **A)** *R. rickettsii* genomic copies quantified in whole blood at selected days post-infection by qPCR targeting the *sca4* gene. Unvaccinated dogs exhibited high and sustained bacteremia across all challenges. Vaccinated groups independent of antigen sources fro Iowa, Morgan or Sheila Smith showed significantly reduced bacterial loads, with many animals presenting PCR values near or below the detection limit. **B)** *R. rickettsii* genomic copies quantified in selected tissue samples at the terminal time points. Pathogen DNA assessed similarly in tissue samples. Infection controls showed detectable to high percentage of bacterial loads, particularly after Morgan and Sheila Smith challenges. In contrast, all vaccine groups and Iowa controls exhibited markedly reduced bacterial numbers with several animals showing undetectable copies. Red bars = Sheila Smith challenge groups, black = Morgan challenge groups, green = Iowa challenge groups. SS: Sheila Smith, M: Morgan, IA: Iowa. Data represent mean ± SEM; Comparison between the different groups was evaluated by a two-way ANOVA with Tukey’s multiple comparisons test.

**Figure 10.**
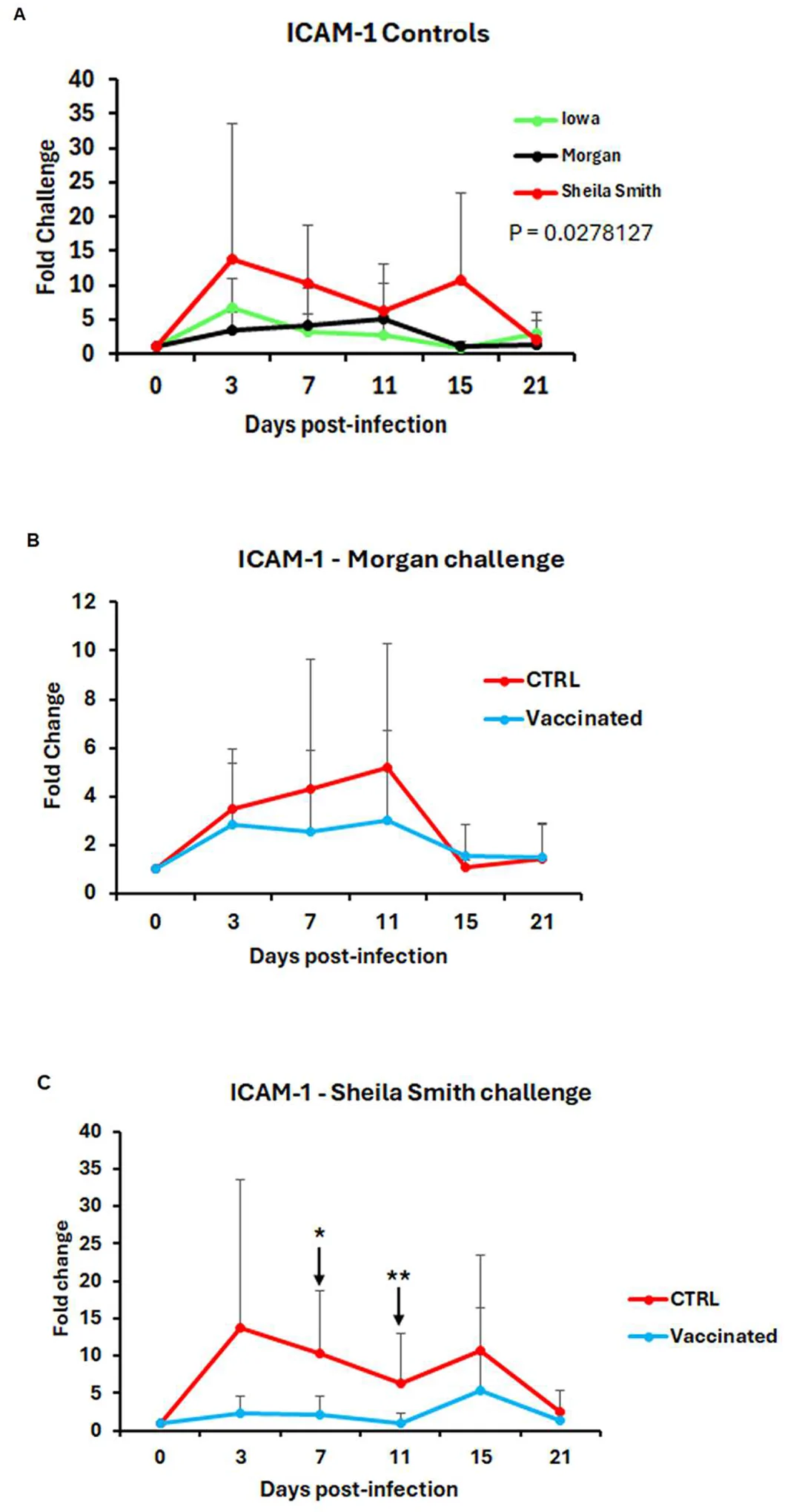
ICAM-1 concentrations following homologous and heterologous *R. rickettsii* challenge. **A)** ICAM-1 levels, evaluated by ELISA, in unvaccinated dogs infected with Sheila Smith, Morgan, or Iowa strains. Sheila Smith controls showed the highest and most sustained level of ICAM-1, and Morgan controls displayed a low rise. Iowa controls had only a transient expression with low-level peak on day 3, returning rapidly to baseline. Panels **B** and **C** refer to ICAM-1 levels in vaccinated dogs following the Morgan and Sheila Smith strain challenges, respectively. All vaccine groups maintained low ICAM-1 concentrations throughout infection with Sheila Smith challenged group showed minimal or no increase compared to unvaccinated controls. Values represent mean ± SEM, A two-way ANOVA with Tukey’s multiple comparisons test was performed to compare the groups.

**Figure 11.**
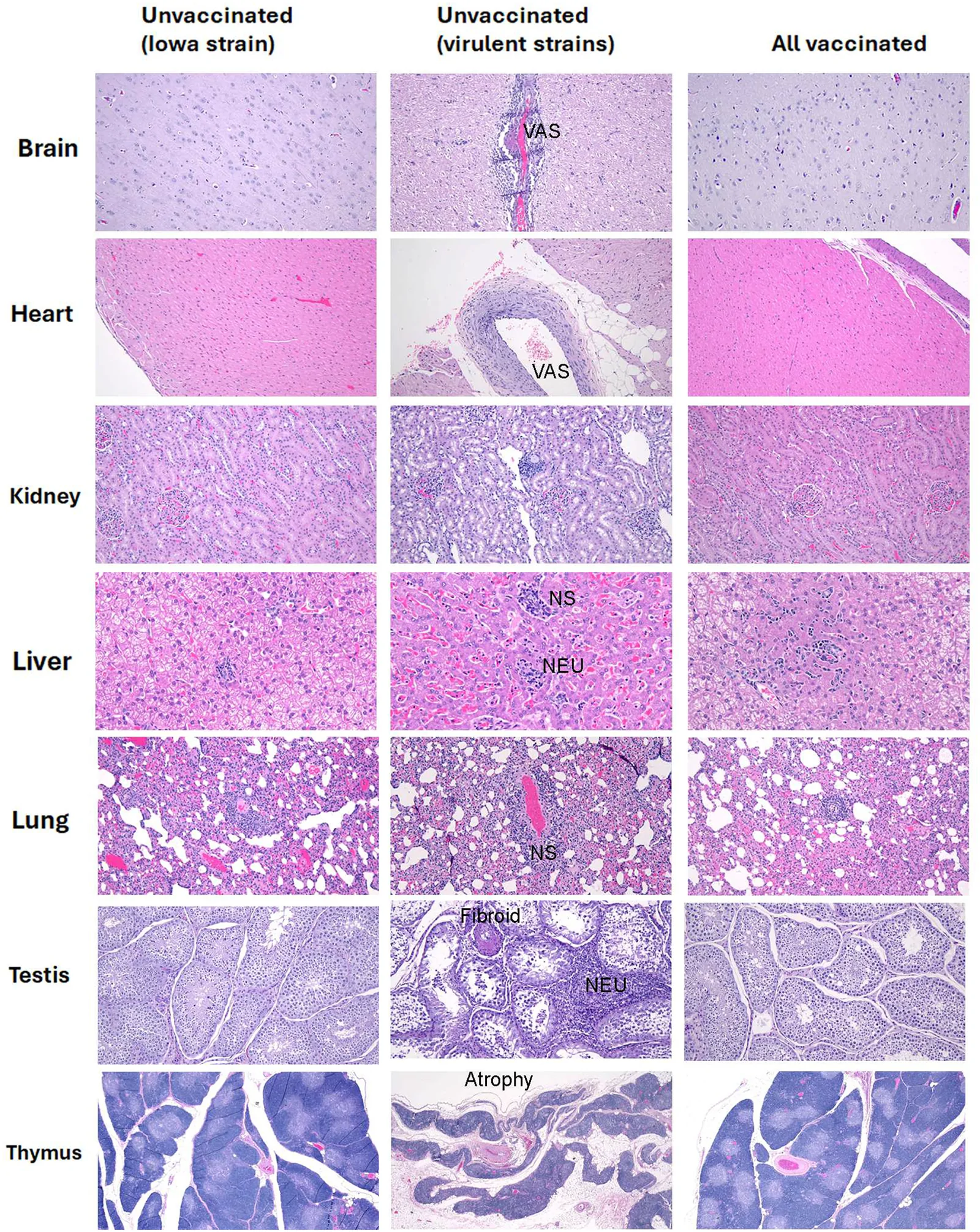
Representative histopathological findings in tissues from vaccinated and unvaccinated dogs following *R. rickettsii* challenge. The represented images of hematoxylin and eosin (H&E)-stained organ sections (tissue sources were identified in the Figure) showing no detectible lesions in unvaccinated controls infected with the avirulent Iowa strain (first column of images) and typical lesions in tissue samples of animals infected with the two virulent strains (Sheila Smith or Morgan) (2^nd^ column of images), whereas minimal evidence of lesions in tissue samples of all vaccinated animals (3^rd^ column of images). Lesions in virulent strain infected unvaccinated dogs included vasculitis (VAS), inflammatory infiltrates like neutrophilic inflammation (NEU), and organ-specific structural damage, whereas all vaccinated dogs exhibited absent or mild (grade 1) lesions with preserving healthy tissue architecture.

**Figure 12.**
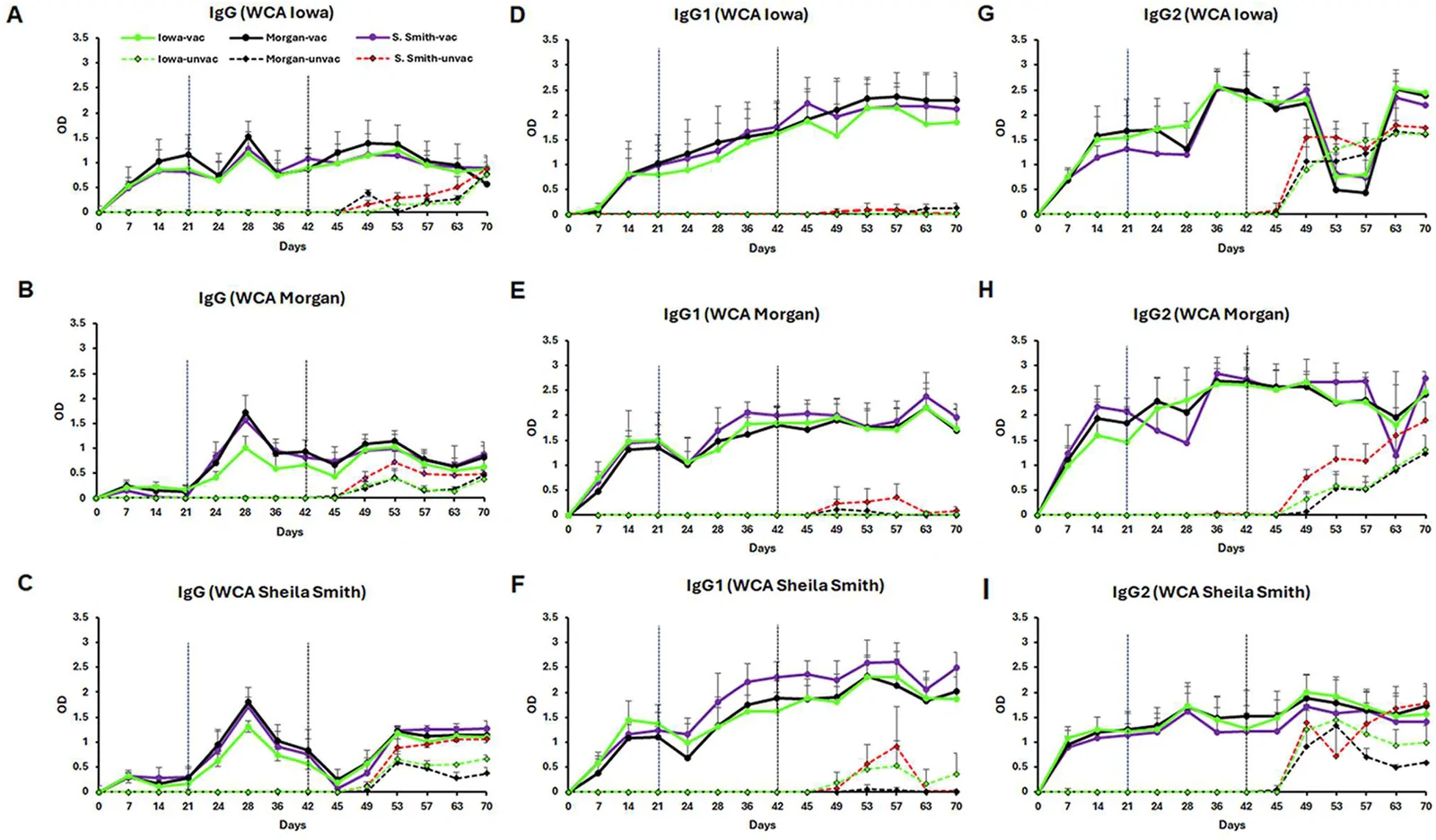
Antigen-specific total IgG and IgG subclass (IgG1 and IgG2) responses in vaccinated and unvaccinated dogs. Plasma from all dogs following mock or WCAV vaccination and following virulent strain infection challenges were analyzed longitudinally for total IgG **(A–C**) and IgG subclass antibodies; IgG1 **(D–F**), and IgG2 (**G–I**) using three whole-cell antigen (WCA) preparations of Iowa, Morgan, or Sheila Smith. All vaccinated groups mounted strong, sustained antibody responses that increased after primary and booster vaccinations and were further boosted following infection challenge (vertical dotted lines). Both IgG isotypes with nearly equal quantities were observed in the vaccinees, whereas IgG2 was the major expressed antibody in infection controls following infection. Values represent mean OD ± SEM.

**Figure 13.**
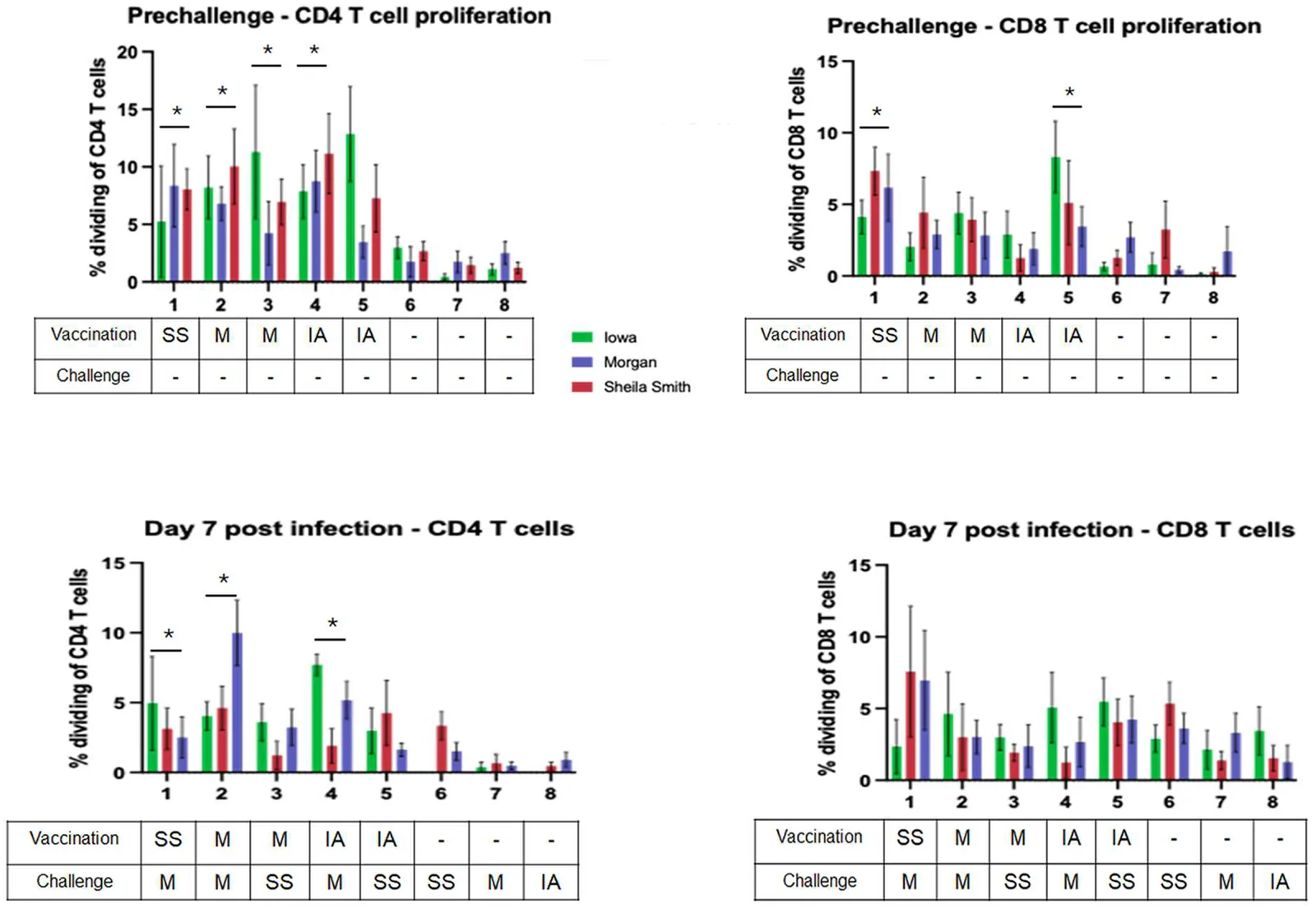
Antigen-specific CD4^+^ and CD8^+^ T cell proliferation in vaccinated and unvaccinated dogs prior to and after *R. rickettsii* infection challenges. PBMCs were stimulated *ex vivo* with whole-cell antigen preparations from Iowa (green), Morgan (blue), or Sheila Smith (red) strains. For each graph, the colored bars represent the proliferation response elicited by the corresponding antigenic stimulation. T cell proliferation was quantified as the percentage of dividing CD4^+^ or CD8^+^ T cells following 5 days of culture. **A** and **B**) Pre-challenge responses of antigen-specific CD4^+^ and CD8^+^ T cell proliferations, respectively, measured at day 42 (prior to infection challenges). **C** and **D**) Day 7 post-infection recall proliferation responses in CD4^+^ and CD8^+^ T cells, respectively, after homologous or heterologous strain infection challenges. The tabular data beneath each panel indicate for each bar cluster (1–8) for the vaccination strains and challenge strains (SS = Sheila Smith; M = Morgan; IA = Iowa; – = unvaccinated or unstimulated). Data represent mean ± SEM. Statistical significance was determined by one-way ANOVA with Tukey’s post-hoc test. *P < 0.05; **P < 0.01.
